# Sex and income inequalities in preventive services in diabetes

**DOI:** 10.1080/13814788.2022.2159941

**Published:** 2023-01-20

**Authors:** Sara Ares-Blanco, Juan A. López-Rodríguez, Mario Fontán Vela, Elena Polentinos-Castro, Isabel del Cura-González

**Affiliations:** a Federica Montseny Health Centre, Gerencia Asistencial Atención Primaria, Servicio Madrileño de Salud, Madrid, Spain; b Medical Specialties and Public Health, School of Health Sciences, University Rey Juan Carlos, Alcorcón, Madrid, Spain; c Instituto de Investigación Sanitaria Gregorio Marañón, Madrid, Spain; d General Ricardos Health Centre, Gerencia Asistencial Atención Primaria, Servicio Madrileño de Salud, Madrid, Spain; e Primary Care Research Unit, Gerencia de Atención Primaria, Servicio Madrileño de Salud, Madrid, Spain; f Health Services Research on Chronic Patients Network (REDISSEC), Health Outcomes-Oriented Cooperative Research Networks (RICORS-RICAPS), ISCIII, Madrid, Spain; g Medicina Preventiva Department, Hospital Universitario Infanta Leonor, Madrid, Spain; h Public Health and Epidemiology Research Group, School of Medicine, Universidad de Alcalá, Madrid, Spain

**Keywords:** Diabetes mellitus, health policy, general practice/family medicine, prevention, surveys

## Abstract

**Background:**

Cancer preventive services (gynaecological cancer screening, colon cancer screening) and cardiometabolic screening are recommended by guidelines to individuals. People with diabetes were less likely to receive them than those without diabetes in some studies.

**Objectives:**

To analyse differences in the coverage of preventive services in people with diabetes compared to non-diabetic individuals and in people with diabetes according to sex and household income.

**Methods:**

We analysed data collected from the European Health Interview Survey 2013–2015, including individuals aged 40–74 (*n* = 179,318), 15,172 with diabetes from 29 countries. The income of a household (HHI) was described in quintiles. The relationship between the coverage of preventive services (cardiometabolic, vaccination, cancer screening) and sociodemographic characteristics was analysed with multiple logistic regression.

**Results:**

Women comprised 53.8% of the total and 40% were 60–74 years. People with diabetes compared to those without diabetes had higher reported coverage of cardiometabolic screening (98.4% vs. 90.0% in cholesterol measurement; 97.0% vs. 93.6% in blood pressure measurement), colorectal cancer screening (27.1% vs. 24.6%) but lower coverage of gynaecological cancer screening (mammography: 29.2% vs. 33.5%, pap smear test: 28.3% vs. 37.9%). Among diabetic patients, women were less likely to receive cholesterol screening (OR = 0.81; 95% CI: 0.72–0.91) and colon cancer screening (OR = 0.79; 95% CI: 0.73–0.86) compared to men. Being affluent was positively associated with receiving cardiometabolic screening and mammography in diabetic patients.

**Conclusion:**

People with diabetes reported higher coverage of preventive services except gynaecological cancer screening. Disparities were found in diabetes among women and less affluent individuals.

KEY MESSAGES

- People with diabetes had higher reported coverage of preventive services except gynaecological cancer screening.
- In people with diabetes, disparities were found among women and less affluent individuals.
- More studies are needed to address preventive services prioritisation in people with diabetes.

## Introduction

Diabetes caused 8.5% of all-cause mortality in Europe in 2018 [1]. At least 32.7 million adults have diabetes in the European Union (EU). The disease prevalence among EU countries differs from 9.9% in Portugal to 3.4% in Ireland.

Diabetes is a complex disease influenced by lifestyle choices and social determinants [2]. Preventive health services (regular exercise, proper diet, and blood pressure monitoring) are recommended not only for diabetes but also for cardiovascular risk factors and cancer screenings in the general population. Colorectal cancer screening starts at 50 years old, although the strategy (faecal blood test or colonoscopy) varies according to the guidelines [2–5]. The European Centre for Disease Prevention and Control (ECDC) recommends influenza vaccination to older age groups and at-risk groups, including people with diabetes [6].

Data from Eurobarometer indicates that health-related behaviours are more frequent in patients with the highest socioeconomic status, women, and older patients [7]. However, the EU Charter of Fundamental Rights states that everyone has the right to preventive care under the conditions established by national laws and practices.

Some studies show that services, such as mammography, Pap smear or colon cancer screenings, are less likely to be performed in people with diabetes than those without diabetes [8–12]. In turn, diabetic patients describe fair or poor self-perceived health (SPH) more frequently than the general population [13]. Preventive care is mainly provided in primary care settings; however, the delivery of these services is influenced by the patient’s attitude, educational level, doctor’s readiness and the practice’s organisation [14–17]. The participation rate also depends on the screening strategies: countries with population-based cancer screening programmes minimised educational inequalities compared to those following an opportunistic testing strategy [17]. Reimbursement may influence coverage of preventive services [18]. The association between reimbursements and socioeconomic inequalities is still unknown [19]. This study aimed 1) to describe differences in preventive services between people with diabetes and people without diabetes in Europe and2) to describe differences in preventive services in people with diabetes according to sex, household income (HHI), and self-perceived health status across countries in Europe.

## Methods

### Study design and population

An observational, cross-sectional survey based on individuals from 40 to 75 years old, who answered the survey in 29 countries, was conducted (*n*: 179,318. Supplement 1: population by country). The European Health Interview Survey (EHIS) is sponsored by the European Commission and is run every five years to obtain data on health from European citizens. Simple randomiszed sampling is conducted to obtain a nationally representative population for each country.

### Data collection

Data was collected between 2013 and 2015. The questionnaires were interviewer-administered, telephone interviews or self-administered. The methodology of the surveys can be checked through Eurostat [20–22].

### Variables

Sociodemographic variables were collected. Household Income (HHI) was the total income of a household after tax deductions according to the household size and the variable was obtained from each country as a quintile (HHI1 quintile belongs to the lowest income household and HHI5 belongs to the more affluent).

Distribution of each preventive service in each country was shared as a quartile’s classification of the percentage of patients who received them. Being Q1 equals the lower quartile and being Q4 equals the highest quartile where the highest proportion of patients received these services.

A healthy diet was collected as eating vegetables and fruits at least 4–6 days a week following. Physical activity was defined as practising exercise for at least 2.5 h per week. Alcohol abuse was described as daily consumption over 20 g in women and 40 g in men daily. Tobacco use was collected as smoking every day. Comorbidities were assembled. Self-perceived health was recorded as responses to the question, ‘How is your health in general?’ The possible answers were very good, good, fair, bad, and very bad.

Preventive services were reviewed in advance to include the target population and frequency according to the latest guidelines and national recommendations (Supplement 2 and 3): Cardiometabolic prevention was recorded as the last year patients had measured their glucose, cholesterol, blood pressure (BP) in people with diabetes and as the last three years in people without diabetes [23]. Cancer screening: mammography and cervical smear tests performed in the last two years (target population: mammography (50–69 years old), Pap smear (40–64 years old). Faecal occult blood test (FOBT) was collected as any test performed in the last two years, and colonoscopy was recorded as any test received throughout life.

Influenza vaccination was assessed as any shot in the last year in patients over age 60 years without diabetes and annually in people with diabetes. Healthcare service use was studied as GP visits in the last year.

### Statistical analyses

Patient characteristics were described using descriptive statistics as proportions. Bivariate analysis with *χ*^2^ test was performed for categorical variables. The relationship between preventive services and patient characteristics was analysed with multiple logistic regression using robust standard errors considering the recruitment was made by countries. The results are presented as odds ratios (ORs) with the corresponding 95% confidence intervals (95% CI). All the tests were conducted at a significance level of 0.05. The analysis was performed using STATA 14.

### Ethics approval and consent to participate

The survey was conducted under Commission Regulation (EU) No 1338/2008 and No 141/2013. Ethical approval was obtained by the national institutions. Rey Juan Carlos University at Madrid authorised the application of EHIS data for health research purposes, and data was provided by Eurostat. The database was anonymised without any personal identifiers. The categories with fewer than 49 individuals were not reported to avoid possible identification.

## Results

A total of 179,318 subjects were included, 53.8% were women and 40.1% of the population was between 60 and 74 years old. The characteristics of all participants are shown in Table 1. 15,172 had diabetes. Compared to people without diabetes, people with diabetes were older (66.4% of them were between 60 and 74 years compared to 37.4%, *p* < 0.001) and more likely to have a lower HHI (21.6% vs. 17%, *p* < 0.001). They had more comorbidities, such as coronary heart disease (15.2%) and chronic renal disease (8.2%). They described their health as bad or very bad more frequently (24.9% vs. 7.7% of non-diabetic, *p* < 0.001).

**Table 1.** Characteristics of the study sample and preventive services by having or not having diabetes.

Patient characteristics:	All (*n*, %)	People without diabetes (*n*, %)	People with diabetes (*n*, %)	*p* ValueA
*N*	179,318	161,370	15,172	
Age				
40–49 years	53,042 (29.6)	51,090 (31.7)	1385 (9.1)	<0.001
50–59 years	54,429 (30.4)	49,917 (30.9)	3709 (24.4)	
60–74 years	71,847 (40.1)	60,363 (37.4)	10,078 (66.4)	
Women	96,431 (53.8)	87,596 (54.3)	7353 (48.5)	<0.001
Educational level				
Low	25,569 (14.3)	21,024 (13.0)	4042 (26.6)	<0.001
Middle	108,121 (60.3)	97,814 (60.6)	8792 (57.9)	
High	45,628 (25.4)	42,532 (26.4)	2338 (15.4)	
Household income				
1	29,269 (17.4)	25,649 (17.0)	3075 (21.6)	<0.001
2	32,007 (19.1)	28,036 (18.6)	3399 (23.9)	
3	33,919 (20.2)	30,368 (20.1)	3015 (21.2)	
4	35,669 (21.2)	32,543 (21.5)	2634 (18.5)	
5	37,073 (22.1)	34,468 (22.8)	2099 (14.8)	
Self-perceived health				<0.001
Very good and good	109,989 (62.9)	104,336 (66.3)	4255 (28.7)	
Fair	48,790 (27.9)	41,031 (26.1)	6871 (46.4)	
Bad and very bad	16,057 (9.2)	12,114 (7.7)	3681 (24.9)	
Lifestyle factors				
Healthy diet	116,614 (65.0)	105,162 (65.2)	9853 (64.9)	0.58
Exercise	175,828 (98.1)	158,102 (98.0)	14,992 (98.8)	<0.001
Tobacco	41,949 (23.7)	38,514 (24.2)	2841 (19.0)	<0.001
Alcohol abuse	5307 (3.2)	4754 (3.2)	458 (3.1)	0.81
Chronic conditions				
Obesity	40,998 (22.9)	33,500 (20.8)	6711 (44.2)	<0.001
Hypertension	51,095 (28.8)	41,144 (25.5)	9176 (60.9)	<0.001
Coronary heart disease	9427 (5.3)	7033 (4.4)	2260 (15.2)	<0.001
Stroke	2620 (1.5)	1974 (1.2)	620 (4.2)	<0.001
Renal disease	5434 (3.1)	4156 (2.6)	1217 (8.2)	<0.001
Preventive services				
Cholesterol measurement^†^	159,717 (90.8)	142,409 (90.0)	14,924 (98.4)	<0.001
BP measurement^†^	164,619 (93.9)	147,547 (93.6)	14,722 (97.0)	<0.001
Influenza vaccination^‡^	26,179 (34.0)	19,120 (31.7)	6178 (40.7)	<0.001
Faecal occult blood testing^§^	31,412 (24.9)	27,085 (24.6)	3742 (27.1)	<0.001
Colonoscopy^¶^	39,442 (31.2)	33,418 (30.3)	4801 (34.8)	<0.001
Mammography^§^	36,417 (34.2)	31,543 (33.5)	3036 (29.2)	<0.001
Cervical smear test^#^	50,016 (37.3)	47,083 (37.9)	2289 (28.3)	<0.001
Health care services				
GP visit^†^	134,609 (75.4)	118,658 (73.8)	13,906 (91.9)	<0.001

A: *p*-Values obtained *via* chi-square tests. ^†^Measurement in the last 5 year in general population and in the last year in people with diabetes. ^‡^Any shot in the last year in patients over 60 years and annually in people with diabetes ^§^measurement biennial. ^¶^At least once in life. ^#^Once every three years.

### Preventive services in people with diabetes vs. people without diabetes

Cardiometabolic screening was more frequent in people with diabetes than in the general population according to the guidelines in both groups (cholesterol measurement: 98.4% vs. 90.0%. BP measurement: 97% vs. 93.6%, *p* < 0.001). Participation in colon cancer screening was slightly higher in people with diabetes (FOBT: 27.1% vs. 24.6%, *p* < 0.001. Colonoscopy: 34.8% vs. 30.3%, *p* < 0.001). In gynaecological cancer screening, women with diabetes were less likely to receive Pap smear (28.3% vs. 37.9%, *p* < 0.001) and mammography (29.2% vs. 33.5%, *p* < 0.001) than those without diabetes.

### Preventive services in people with diabetes regarding country, HHI and sex

**Figure 1.**
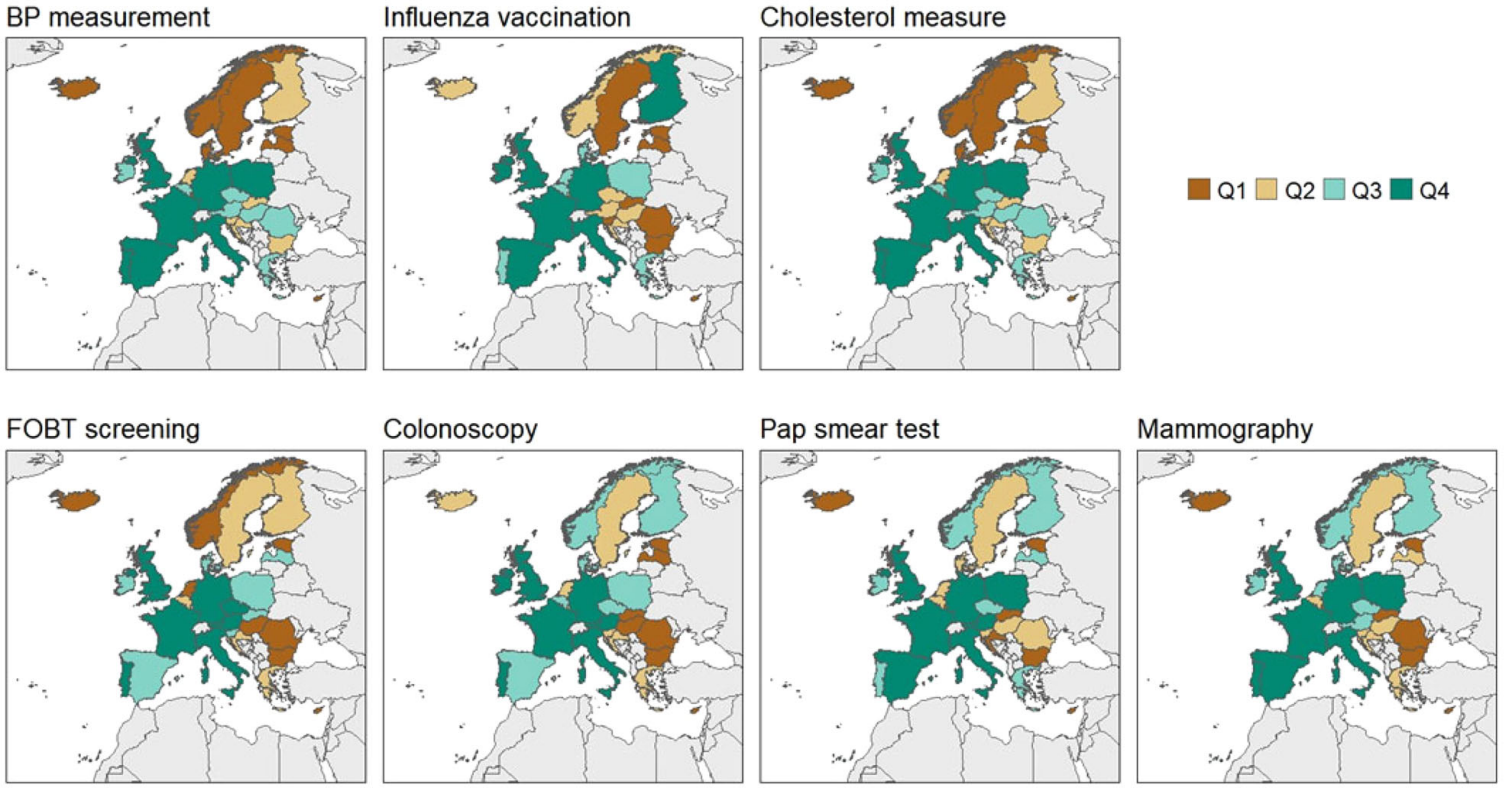
Reported coverage of all preventive services for people with diabetes in Europe. The proportion of patients who received each preventive service was divided in quartiles classification by countries. Being Q1 equals to the lower quartile, meaning that the lowest proportion of coverage of the service was described in these countries. Being Q2 equals to the second quartile, meaning that the next lowest proportion of the service was observed in these countries. Being Q3 equals to the third quartile, meaning that the next highest proportion of the service was described in these countries. Being Q4 equals to the fourth quartile, meaning that the highest proportion of the service was observed in these countries.

The reported coverage of preventive services in people with diabetes varied across Europe (Figure 1 and Supplement 4). Table 2 shows differences between different HHI groups among women and men with diabetes. Women who belong to HHI 2-4 groups had higher percentage of cholesterol and BP measurements compared to women in HH1 and HHI5 groups (*p* < 0.001 and *p* < 0.009, respectively). Fewer mammography and Pap smears were performed in women who belonged to HHI 1 than HHI 5 (mammography: 54.3% vs. 67.7%, *p* < 0.001; cervical smear test: 50.7% vs. 66.9%, *p* < 0.001). Men with the lowest HHI had their cholesterol and BP measured less frequently. The cholesterol measurement gradually improved their reported coverage as men belong to the higher HHI (*p* > 0.001). Men who belong to HHI 2–4 groups had their BP measured more frequently than those in HHI1 group and HHI5 groups (*p:* 0.019). These same men received less frequent colon screening than those with the highest HHI but no significance was found (*p:* 0.14).

**Table 2.** Preventive services, chronic conditions and GP visits of people with diabetes regarding sex and household income.

	HHI 1 (less affluent)	HHI 2	HHI 3	HHI 4	HHI 5 (more affluent)	*p* ValueA
Women*	1677 (24.3)	1812 (*n*:26.2)	1446 (20.9)	1174 (17.0)	788 (11.4)	
Age						
40–49 years	190 (30.7)	145 (23.4)	99 (16.0)	102 (16.5)	82 (13.2)	<0.001
50–59 years	453 (27.7)	346 (21.1)	289 (17.6)	294 (17.9)	253 (15.4)	
60–74 years	1034 (22.2)	1321 (28.4)	1058 (22.7)	778 (16.7)	453 (9.7)	
Chronic conditions						
Obesity	866 (51.6)	892 (49.2)	680 (47.0%)	523 (44.5)	324 (41.1)	<0.001
Hypertension	1098 (65.9)	1186 (65.8)	933 (65.0%)	721 (61.8)	430 (55.2)	<0.001
Coronary heart disease	272 (16.6)	270 (15.2)	216 (15.1%)	144 (12.5)	76 (9.8)	<0.001
Stroke	74 (4.5)	72 (4.1)	53 (3.7%)	20 to 49 observations	20 to 49 observations	0.11
Renal disease	170 (10.4)	175 (9.9)	115 (8.1%)	86 (7.5)	20 to 49 observations	<0.001
Preventive Services						
Cholesterol measurement^†^	1426 (85.0)	1583 (87.4)	1273 (88.0)	1049 (89.4)	656 (83.2)	<0.001
BP measurement^†^	1518 (90.5)	1672 (92.3)	1357 (93.8)	1087 (92.6)	717 (91.0)	0.009
Influenza vaccination^†^	649 (38.7)	733 (40.5)	554 (38.3)	446 (38.0)	322 (40.9)	0.48
Faecal occult blood test^‡^	418 (28.1)	430 (25.8)	339 (25.2)	280 (26.1)	184 (26.1)	0.45
Colonoscopy^¶^	506 (34.0)	529 (31.7)	427 (31.7)	372 (34.7)	230 (32.6)	0.36
Mammography^‡^	600 (54.3)	704 (59.5)	603 (64.1)	530 (62.9)	398 (67.7)	<0.001
Cervical smear test^#^	379 (50.7)	377 (55.0)	334 (60.2)	343 (62.1)	283 (66.9)	<0.001
GP visit^†^	1564 (93.7)	1689 (93.5)	1354 (93.8)	1083 (92.3)	701 (89.1)	<0.001
Men*	1398 (19.0)	1587 (21.6)	1569 (21.4)	1460 (19.9)	1311 (17.9)	<0.001
Age						
40–49 years	172 (25.0)	120 (17.4)	145 (21.0)	117 (17.0)	134 (19.4)	
50–59 years	410 (22.2)	341 (18.5)	336 (18.2)	375 (20.3)	381 (20.6)	
60–74 years	816 (17.0)	1126 (23.4)	1088 (22.7)	968 (20.1)	796 (16.6)	
Chronic conditions						
Obesity	625 (44.7)	684 (43.1)	643 (41.0)	542 (37.1)	503 (38.4)	<0.001
Hypertension	846 (61.3)	938 (59.8)	912 (58.4)	839 (57.7)	715 (55.0)	0.015
Coronary heart disease	244 (18.1)	308 (20.0)	246 (15.9)	205 (14.3)	147 (11.4)	<0.001
Stroke	80 (6.0)	86 (5.6)	71 (4.6)	53 (3.7)	20 to 49 observations	<0.001
Renal disease	144 (10.6)	134 (8.7)	122 (8.0)	96 (6.7)	64 (5.0)	<0.001
Preventive Services						
Cholesterol measurement	1181 (84.5)	1372 (86.5)	1375 (87.6)	1299 (89.0)	1177 (89.8)	<0.001
BP measurement^†^	1253 (89.6)	1428 (90.0)	1445 (92.1)	1350 (92.5)	1200 (91.5)	0.019
Influenza vaccination^†^	569 (40.7)	735 (46.3)	684 (43.6)	625 (42.8)	563 (42.9)	0.041
Faecal occult blood test^‡^	346 (28.2)	386 (26.3)	431 (30.3)	398 (29.6)	348 (29.6)	0.14
Colonoscopy^¶^	437 (35.6)	526 (35.9)	517 (36.3)	528 (39.3)	458 (38.9)	0.14
GP visit^†^	1261 (90.6)	1432 (90.6)	1416 (90.4)	1333 (91.4)	1169 (89.4)	0.49

HHI: household income A: *p*-Values obtained *via* chi-square tests *: *n*, % ^†^Measurement in the last year ^‡^measurement biennial. ^¶^At least once in life ^#^Once every three years.

Factors associated with the compliance of each preventive service in people with diabetes are shown in Table 3; these factors are also described separately by sex in Supplement 5–6. Women were less likely to receive cholesterol screening (adjusted OR (aOR)=0.85; 95% CI: 0.76–0.95), influenza vaccination (aOR = 0.80; 95% CI: 0.74–0.86) and colon cancer screening (colonoscopy aOR = 0.82; 95% CI: 0.75–0.88). Being in more affluent HHI groups was positively associated with receiving cardiometabolic screening and mammography.

**Table 3.** Results of the multiple logistic regression to describe factors associated with the compliance of each preventive service in people with diabetes (*n* = 15,172).

	Model 1: Cholesterol measurement OR (95% CI)	Model 2: BP measurement OR (95% CI)	Model 3: Influenza vaccination OR (95% CI)	Model 4: Faecal occult blood testing OR (95% CI)	Model 5: Colonoscopy OR (95% CI)	Model 6: Cervical smear test OR (95% CI) (*n* = 2,289)	Model 7: Mammography OR (95% CI) (*n* = 3,036)
Sex (male: reference)	0.85 (0.76–0.95)	0.99 (0.87–1.14)	0.80 (0.74–0.86)	0.87 (0.81–0.95)	0.82 (0.75–0.88)		
Age							
40–44years	Reference	Reference	Reference			Reference	
45–49 years	1.49 (1.10–2.02)	1.04 (0.71–1.52)	1.39 (1.05–1.83)			0.82 (0.64–1.06)	
50–54 years	1.80 (1.36–2.39)	1.30 (0.91–1.87)	1.53 (1.18–1.98)	Reference	Reference	0.77 (0.61–0.97)	Reference
55–59 years	2.11(1.61–2.78)	1.36 (0.96–1.92)	1.42 (1.11–1.83)	1.03 (0.87–1.20)	1.08 (0.92–1.26)	0.64 (0.51–0.80)	1.02 (0.82–1.27)
60–64 years	1.89 (1.46–2.46)	1.38 (0.98–1.93)	1.76 (1.37–2.25)	1.18 (1.02–1.37)	1.43 (1.24–1.66)	0.52 (0.42–0.65)	0.82 (0.66–1.01)
65–69 years	2.10 (1.62–2.73)	1.50 (1.07–2.10)	2.76 (2.16–3.59)	1.15 (0.99–1.34)	1.44 (1.24–1.66)		0.55 (0.45–0.67)
70–74 years	1.88 (1.44–2.44)	1.55 (1.10–2.18)	3.23 (2.53–4.12)	1.03 (0.89–1.20)	1.64 (1.42–1.90)		
Educational level (low: reference)							
Middle	1.10 (0.89–1.15)	1.31 (1.12–1.54)	0.78 (0.72–0.85)	1.33 (1.21–1.46)	1.16 (1.06–1.27)	1.03 (0.90–1.18)	0.70 (0.60–0.81)
High	1.08 (0.89–1.30)	1.55 (1.22–1.96)	1.22 (1.08–1.38)	1.41 (1.23–1.61)	1.70 (1.50–1.94)	0.98 (0.81–1.18)	0.77 (0.61–0.97)
HHI: (1: reference)							
2	1.23 (1.05–1.44)	1.20 (0.99–1.46)	1.06 (0.95–1.19)	0.88 (0.78–0.99)	0.92 (0.82–1.03)	1.06 (0.91–1.24)	1.22 (1.03–1.46)
3	1.24 (1.06–1.46)	1.48 (1.20–1.82)	0.95 (0.85–1.06)	0.91 (0.80–1.03)	0.96 (0.85–1.08)	0.94 (0.80–1.11)	1.47 (1.21–1.77)
4	1.49 (1.25–1.78)	1.48 (1.19–1.84)	0.98 (0.87–1.10)	0.92 (0.81–1.05)	1.11 (0.98–1.25)	0.96 (0.81–1.13)	1.36 (1.11–1.65)
5	1.25 (1.04–1.51)	1.33 (1.05–1.67)	0.98 (0.86–1.12)	0.88 (0.77–1.02)	1.00 (0.87–1.14)	0.83 (0.70–1.00)	1.59 (1.26–2.01)
SPH: (Very good and good: reference)							
Fair	1.21(1.06–1.37)	1.40 (1.20–1.63)	0.87 (0.79–0.95)	1.04 (0.95–1.15)	1.14 (1.01–1.22)	1.07 (0.95–1.21)	0.86 (0.73–1.01)
Bad and very bad	1.18 (1.91–1.39)	1.63 (1.33–2.01)	0.81 (0.73–0.90)	1.01 (0.90–1.14)	1.24 (1.11–1.40)	1.08 (0.92–1.26)	0.71 (0.59–0.86)
Lifestyle factors							
Healthy diet	1.27 (1.13–1.42)	1.10 (0.95–1.27)	1.24 (1.15–1.34)	1.125 (1.06–1.26)	1.03 (0.95–1.12)	1.64 (1.47–1.84)	1.54 (1.35–1.76)
Exercise	0.75 (0.44–1.29)	0.94 (0.50–1.76)	1.52 (1.07–2.15)	0.83 (0.58–1.18)	1.22 (0.85–1.76)	1.32 (0.82–2.13)	0.78 (0.40–1.53)
Tobacco	0.88 (0.77–1.01)	0.83 (0.70–0.98)	0.78 (0.71–0.86)	0.83 (0.74–0.93)	0.86 (0.77–0.95)	0.67 (0.59–0.76)	0.94 (0.79–1.12)
Chronic conditions							
Obesity	1.27 (1.13–1.42)	1.33 (1.15–1.54)	1.09 (1.01–1.18)	1.01 (0.93–1.09)	1.06 (0.98–1.15)	1.06 (0.95–1.18)	1.15 (1.01–1.30)
Hypertension	0.82 (0.73–0.92)	0.54 (0.47–0.62)	0.97 (0.90–1.05)	1.00 (0.92–1.09)	1.06 (0.98–1.15)	0.96 (0.86–1.07)	1.07 (0.94–1.23)
Coronary heart disease	1.44 (1.20–1.73)	1.54 (1.19–2.00)	0.88 (0.79–0.98)	0.95 (0.84–1.06)	0.94 (0.84–1.05)	0.69 (0.57–0.82)	0.84 (0.69–1.01)
Stroke	1.01 (0.75–1.36)	0.92(0.60–1.41)	1.05 (0.87–1.27)	1.03 (0.84–1.26)	1.12 (0.93–1.37)	1.31 (0.95–1.79)	1.30 (0.93–1.80)
Renal disease	0.68 (0.54–0.86)	0.54 (0.37–0.77)	0.95 (0.83–1.09)	0.896(0.74–0.99)	0.78 (0.67–0.89)	0.82 (0.67–1.00)	0.76 (0.60–0.96)

OR: odds ratio; CI: confidence interval.

## Discussion

### Main findings

People with diabetes had higher reported coverage of cardiometabolic preventive services and colon cancer screening than those without diabetes but women with diabetes received suboptimal gynaecological cancer screening than those without diabetes. An association between being a diabetic woman and receiving fewer preventive care services (cholesterol measurement, influenza vaccination and colorectal cancer screening) was found. Those diabetic subjects with bad or very bad self-perceived health were more likely to receive cardiometabolic services and colonoscopy screening, but they received less influenza vaccination and less mammography.

### Comparison with existing literature

Our results are concordant with a meta-analysis on cancer screening in a diabetic population [8]. They found that women with diabetes received fewer colon cancer screenings and that having diabetes was associated with a lower likelihood of gynaecological cancer screening than not having diabetes [20]. In addition, other factors must be considered. People with diabetes were older than people without diabetes; being an elderly adult could be related to receiving more cardiometabolic screening. A report from the CDC recognised that a quarter of individuals from 50 to 64 years received preventive care compared to half of individuals over 65 years [24]. Patients with comorbidities (including diabetes) were less likely to undergo mammography and Pap smear than those without comorbidities [25]; these differences could be related to competing health priorities.

People with diabetes said they had fair, bad or very bad self-perceived health more frequently than those without diabetes. Our results are concordant with an annual telephone survey in the United States, where adults with diabetes reported having fair or poor health three times the rate of those without diabetes (46.7% vs. 14.2%) [26]. In this study, women and those in the less affluent quintile (HHI 1) reported worse self-perceived health than men and other HHI groups. These findings are in keeping with others reporting that women and those with lower education have worse self-perceived health in the European context [27].

Diabetic patients who lived in Western Europe achieved better-reported coverage of the preventive service. Likely, the organisation of the health care system in countries with universal coverage could explain higher coverage of preventive services [9,18]. We should consider that the approach to cancer screenings differs from opportunistic to population-based screening in the EU and from regional to national screening [15,19,20], which could explain the differences in participation among the countries. Individuals with diabetes from a population-based breast cancer screening programme were more likely to undergo mammography than those in an opportunistic programme (42.1% vs. 14.1%) in France [21]. In systems with a fee for services, women were less likely to receive preventive services [28].

Factors associated with the compliance of each preventive service in people with diabetes are different. Women received more BP measurements but fewer cholesterol tests, colon cancer screenings and influenza immunisations, which is concordant with the findings of Bhatia et al. [8]. We could not find studies that described the differences in preventive services participation across sexes according to HHI but the evidence supports that more disadvantaged patients are less likely to report lifestyle factors, cardiometabolic tests and influenza vaccinations [29].

Recently, it has been suggested that there must be a priority list of evidence-based preventive services and that list should be discussed with patients [30,31]. We should not forget that comorbidities influenced the number of preventive services patients received [32]; patients with more than two comorbidities received more cardiometabolic preventive services but less cancer screening. Regarding people with diabetes, Taksler et al. suggested that control of hypertension, dyslipidaemia, and diabetes should be prioritised, while cancer screening could be delayed [31]. Although some prioritisation could be recommended, differences due to gender and HHI should be considered to reduce inequities in receiving preventive services.

### Strength and limitations

A strength of our study is that we showed the participation in preventive services of individuals across Europe and described how the HHI influenced the performance of preventive services for men and women with diabetes. Several limitations should be kept in mind when considering these results. All information came from self-reported interviews. Some data could be under-represented, especially in those activities that are not performed annually. We did not differentiate between type 1 and type 2 diabetes since this information was not available in EHIS data. The survey did not gather the family history, the years of evolution of diabetes or the treatment´s patients were receiving. The different methods to collect information could also be a limitation, especially in countries with a self-administered mode.

We compared European countries with different national health systems, cancer screening programmes and lifestyles; thus, our comparisons are limited by the national characteristics of the countries where patients lived. The characteristics of the practices (number of GPs, number of other healthcare professionals, location of the practice – urban or rural) and the type of reimbursement in providing preventive care were not publicly available in all countries, so we could not analyse the results under this perspective.

This study collects information regarding EHIS wave 2 and not pertaining to EHIS wave 3 (survey in 2019–2021) for several reasons. First, most of preventive care services happened in primary care but during the pandemic, the organisation of primary care changed significantly. COVID-19 care was prioritised beyond preventive care. The delivery of preventive care also depended on the COVID-19 waves, which differed in each country. Second, cancer screening and cardiometabolic screening decreased through the pandemic. Third, Eurostat gives national data in different years. Countries surveyed in 2019 would not be comparable to those surveyed in 2020 and 2021; so, we collected EHIS wave 2 to answer this study aims, thinking wave 2 was more reflective of normal practice.

### Implications for clinical practice

Public policies should strengthen primary care to improve preventive coverage in Europe, especially to those groups who receive them less so inequalities in preventive services can diminish. More studies are needed to address preventive services prioritisation in people with diabetes.

### Conclusion

People with diabetes received cardiometabolic and colorectal screenings more frequently than the general population in Europe. Diabetic women were less likely to receive gynaecological cancer screening than non-diabetic women. Among people with diabetes, being a woman was associated with receiving less cholesterol screening, influenza vaccination and colon cancer screening, and being affluent was positively associated with receiving more cardiometabolic screening and more mammography. Having bad or very bad self-perceived health was not only associated with less cardiometabolic services and colonoscopy testing but with not receiving mammography.

## Supplementary Material

Supplemental Material
