# Supplementary material for: Sex and income inequalities in preventive services in diabetes
Source: Eur J Gen Pract. 2023 Jan 20;29(1):2159941. doi: 10.1080/13814788.2022.2159941 (PMC9870013; doi:10.1080/13814788.2022.2159941)
Supplement: Supplemental Material [file IGEN_A_2159941_SM4566.docx]

**Supplement 1:** Population by country.

|  | **Country population in 2015** | | **Study population** | |
| --- | --- | --- | --- | --- |
|  | **Total population (Million)** | **% Of population aged 65+ years** | **Whole population** | **People with diabetes** |
| N | 512.27 |  | 179,318 | 5,172 |
| Austria | 8.50 | 18.8 | 10,034 | 552 |
| Belgium | 11.25 | 18.1 | 4,780 | 370 |
| Bulgaria | 7.02 | 20.1 | 3,791 | 337 |
| Croatia | 10.53 | 18.9 | 3,035 | 2,06 |
| Cyprus | 0.85 | 12.8 | 2,647 | 257 |
| Czech Republic | 10.51 | 18.1 | 3,877 | 474 |
| Denmark | 5.65 | 19.0 | 3,625 | 236 |
| Estonia | 1.31 | 18.8 | 2,967 | 238 |
| Finland | 5.45 | 20.3 | 3,776 | 348 |
| France | 65.83 | 18.9 | 8,879 | 973 |
| Germany | 81,17 | 21.1 | 14,267 | 1,149 |
| Greece | 10.90 | 19.9 | 4,648 | 539 |
| Hungary | 9.87 | 17.5 | 3,055 | 349 |
| Iceland | 0.32 | 13.7 | 2,095 | 116 |
| Ireland | 4.60 | 13.2 | 6,901 | 391 |
| Italy | 60.78 | 22.4 | 14,195 | 982 |
| Latvia | 2.00 | 19.3 | 3,844 | 254 |
| Luxembourg | 0.54 | 14.0 | 2,222 | 162 |
| Malta | 0.42 | 18.4 | 2,392 | 309 |
| Norway | 5.10 | 16.3 | 4,564 | 253 |
| Poland | 38.01 | 15.6 | 13,331 | 1,236 |
| Portugal | 10.42 | 20.7 | 10,571 | 1,354 |
| Romania | 19.94 | 17 | 9,565 | 637 |
| Slovakia | 5.41 | 14.1 | 2,971 | 312 |
| Slovenia | 2.06 | 18.0 | 3,366 | 290 |
| Spain | 46.51 | 18.9 | 13,092 | 1,126 |
| Sweden | 9.64 | 18.0 | 3,190 | 163 |
| The Netherlands | 16.82 | 17.9 | 4,423 | 308 |
| United Kingdom | 64.35 | 15.9 | 13,215 | 1,145 |

**Supplement 2:** Cancer screening according to the latest national recommendations and guidelines (data was collected from official national websites for professionals and patients across Europe in 2021).

|  | Colorectal cancer Screening | | | Mammography | | Cervical smear test | |
| --- | --- | --- | --- | --- | --- | --- | --- |
| Country | **Target Population** | **FOBT Frequency** | **Alternative: Colonoscopy, Frequency** | **Target Population** | **Frequency** | **Target Population** | **Frequency** |
| Austria | No universal screening | | | 45-69  40-44 and >70 | Biennial  Voluntary screening | >18 | Annually* |
| Belgium | 50-74 | Biennial |  | 50-69 | Biennial | 25-64 | 3 years |
| Bulgaria | No universal screening | | | 50-69 | No universal screening | 25-64 | No universal screening |
| Croatia | 50-74 | Biennial |  | 50-69 | Biennial | 25-64 | 3 years |
| Cyprus | 50-69 | No universal screening*** | | 50-69 | Biennial | 24-65 | 2 years |
| Czech Republic | 50-54  >55 | Annually  Biennial |  | **≥**45 | Biennial | >15 | Annually* |
| Denmark | 50-74 | Biennial |  | 50-69 | Biennial | 23-64 | 23-49: 3 years  50-64: 5 years |
| Estonia | 60-69 | No universal screening*** | | 50-65 | Biennial | 30-55 | 5 years |
| Finland | 60-68** | Biennial |  | 50-69 | Biennial | 30-60 | 5 years |
| France | 50-74 | Biennial |  | 50-74 | Biennial | 25-65 | 3 years |
| Germany | 50-74 | 50-54 annually  >54: Biennial or Colonoscopy every 10 years | Women can choose colonoscopy if they are ≥55 years  Men can choose between colonoscopy if they are ≥50 years. | 50-69 | Biennial | 20- 60 | 20-35: Annually  ≥35: every 3 years |
| Greece | No universal screening | | | 49-50** | Biennial | 20-69 | Annually |
| Hungary | 50-70 | Biennial |  | 45-65 | Biennial | 25-65 | 3 years |
| Iceland | No universal screening | | | 40-69 | Biennial | 23-65 | 3 years |
| Ireland | 60-69 | Biennial |  | 50-69 | Biennial | 25-65 | 25-29 years: every 3 years  30-65 years: every 5 years |
| Italy | 50-70 | Biennial |  | 50-69  Tuscany region. 45-74 | Biennial | 25-64 | 3 years |
| Latvia | No universal screening | | | 50-69 | Biennial | 25-70 | 3 years |
| Lithuania | 50-74 | Biennial |  | 50-69 | Biennial | 25-60 | 3 years |
| Luxembourg | 55-74 | Biennial |  | 50-69 | Biennial | >15 | Annually* |
| Malta | 56-70 | Biennial |  | 50-69 | 3 years | 27-39** | 3 years |
| Norway | 50-74 | No universal screening*** | | 50-69 | Biennial | 25-69 | 3 years |
| Poland | 55-64 |  | Once in life | 50-69 | Biennial | 25-59 | 3 years |
| Portugal | No universal screening | | | 50-69 | Biennial | 25-64 | 3 years |
| Romania | No universal screening | | | No universal screening | | 25-64 | 5 years |
| Slovakia | 50-75 | Biennial | Patients can choose colonoscopy instead of FOBT | 40-69 | Biennial | 23-64 | 3 years |
| Slovenia | 50-74 | Biennial |  | 50-69 | Biennial | 20-64 | 3 years |
| Spain | 50-69 | Biennial |  | 50-69 | Biennial | 25-65 | 3 years |
| Sweden | 60-69 | No universal screening*** | | 40-74 | Biennial | 23-64 | 23-50: 3 years  51-64: every 7 years |
| The Netherlands | 55-75 | Biennial |  | 50-75 | Biennial | 30-60 | 5 years |
| United Kingdom | 60-74 | Biennial |  | 50-71 | Every 3 years | 25-64 | - 25-49 every 3 years  - 50 to 64 every 5 years |
| Organizations |  | | | | | | |
| US Preventive Services Task Force | 50-75 | Annual | Once every 10 years in case colonoscopy was performed instead of FOBT | 50-75 | Biennial | 21-65 | Every 3 years |
| Canadian Task Force on Preventive Health Care | 50-74 | Biennial | Sigmoidoscopy every 10 years. | 50-74 | Every 2-3 years | 25-69 | Every 3 years |
| EU Cancer Screening | 50-74 |  |  | 50-69 |  | Starting at 20-30 |  |
| Spanish Preventive Services (PAPPS) | 50-74 | Annual or Biennial | Once every 15 years in case colonoscopy was performed instead of FOBT | 50-69 | Biennial | 25-65 | Every 3 years |

* In the course of preventive health check-ups, general practitioners or gynaecologist can make a prescription for a smear test annually.

**Screening programmes have recently started; they have been launched to begin in a target group to be continued as patients get older.

***No universal screening but pilot programmes or regional screenings are taking place.

**Supplement 3:** Cardiometabolic screening frequency and target population according to the latest guidelines.

|  | Glucose measurement | | Cholesterol measurement | | BP measurement | |
| --- | --- | --- | --- | --- | --- | --- |
|  | **Target populations**  **(years)** | **Frequency** | **Target populations**  **(years)** | **Frequency** | **Target populations**  **(years)** | **Frequency** |
| Organizations |  |  |  |  |  |  |
| US preventive Services Task Force | 40-70 years who are obese | 3 years | 40-75 | Universal linked to cardiovascular risk score calculation | 18-39  ≥ 40  Obese | 3-5 years  Annual  Annual |
| Canadian Task Force on Preventive Health Care | Not routinely screening if low risk |  | No recent recommendation | | ≥18 | At all appropriate primary care visits |
| NICE | 40-74 | 5 years | ≥ 40 | Universal linked to cardiovascular risk score calculation | ≥ 40 | Every 5 years |
| Spanish Preventive Services (PAPPS) | Not described | Every 4 years | ≥ 18 | Every 4 years | ≥ 40 | Every 3-5 years |
| European Society of Cardiology (ESC) |  |  | Men >40  Women >50 | Every 5 years linked to cardiovascular risk score calculation | Men >40  Women >50 | Every 5 years linked to cardiovascular risk score calculation |
| Comorbidities Follow-up | **Blood test frequency** | | | | **BP measurement** | |
| Dyslipidaemia ESC | Annually | | | | The frequency is not described but it should be Linked to cardiovascular risk score calculation | |
| Hypertension ESC | At least every 2 years | | | | Every 3-6 months | |
| Ischaemic Heart Disease ESC | Every 2 years | | | | They do not describe the frequency | |
| Stroke, Royal College of Physicians | At least 3 months later than the stroke episode  (They don´t describe the further follow-up) | | | | It should be monitored frequently | |
| Diabetes, ADA | At least two times a year | | | | In every routine clinical visit. | |
| Renal Disease, KDIGO | More frequently than annually | | | | They do not describe the frequency | |

ESC: European Society of Cardiology. ADA: American Association of Diabetes. KDIGO: Kidney Disease Improving Global Outcomes

**Supplement 4:** Preventive services by countries in people with diabetes (n, %).

| **Country** | **Cholesterol measurement** † | **BP measurement** † | **Influenza Vaccination** † | **FOBT screening**‡ | **Colonoscopy screening** ¶ | **Mammography**‡ | **Cervical smear test** # |
| --- | --- | --- | --- | --- | --- | --- | --- |
| Austria | 494 (89.4) | 498 (90.2) | 62 (11.2) | 261 (52.5) | 293 (58.9) | 126 (32.2) | 124 (36.3) |
| Belgium | 314 (84.8) | 325 (87.8) | 212 (57.3) | 20 to 49 observations | 202 (60.8) | 51 (19.2) | 20 to 49 observations |
| Bulgaria | 275 (81.6) | 298 (88.4) | 20 to 49 observations | 20 to 49 observations | 20 to 49 observations | 20 to 49 observations | 20 to 49 observations |
| Croatia | 251 (80.4) | 265 (84.9) | 79 (25.3) | 72 (24.5) | 83 (28.3) | 71 (30.6) | 53 (28..8) |
| Cyprus | 225 (87.5) | 339 (93.0) | 81 (31.5) | less than 20 observations | 59 (25.3) | 20 to 49 observations | 20 to 49 observations |
| Czech Republic | 434 (91.5) | 458 (96.6) | 87 (18.3) | 186 (41.4) | 174 (38.7) | 119 (38.5) | 79 (39.7) |
| Denmark | 215 (91.1) | 219 (92.8) | 111 (47.0) | 78 (36.1) | 83 (38.4) | 55 (37.4) | 20 to 49 observations |
| Estonia | 213 (89.5) | 222 (93.2) | less than 20 observations | 20 to 49 observations | 178 (80.9) | 20 to 49 observations | 20 to 49 observations |
| Finland | 286 (82.1) | 295 (84.7) | 348 (100.0) | 58 (18.1) | 121 (37.9) | 93 (37.0) | 59 (34.1) |
| France | 778 (79.9) | 907 (93.2) | 528 (54.2) | 392 (47.9) | 390 (47.6) | 278 (41.6) | 242 (39.6) |
| Germany | 1078 (93.7) | 1112 (96.7) | 585 (50.9) | 520 (50.8) | 649 (63.4) | 232 (31.1) | 171 (27.8) |
| Greece | 435 (80.7) | 432 (80.1) | 306 (56.7) | 77 (15.4) | 151 (30.2) | 101 (28.8) | 94 (38.0) |
| Hungary | 310 (88.8) | 332 (95.1) | 140 (40.1) | 20 to 49 observations | 65 (20.9) | 78 (32.3) | 60 (27.6) |
| Iceland | 81 (69.8) | 101 (87.0) | 60 (51.7) | less than 20 observations | 57 (54.2) | 20 to 49 observations | 20 to 49 observations |
| Ireland | 343 (87.7) | 361 (92.3) | 263 (67.2) | 69 (19.7) | 197 (56.4) | 58 (22.4) | 54 (24.4) |
| Italy | 845 (86.0) | 862 (87.7) | 366 (37.2) | 294 (35.5) | 279 (30.8) | 181 (26.9) | 142 (28.8) |
| Latvia | 232 (91.3) | 241 (94.8) | 20 to 49 observations | 88 (37.6) | 47 (20.0) | 60 (34.0) | 68 (47.8) |
| Luxembourg | 157 (96.9) | 158 (97.5) | 69 (42.5) | 35 (25.0) | 78 (55.7) | 20 to 49 observations | 20 to 49 observations |
| Malta | 268 (86.7) | 279 (90.2) | 157 (50.8) | 65 (22.5) | 50 (17.3) | 38 (17.4) | 20 to 49 observations |
| Norway | 208 (82.2) | 232 (91.7) | 64 (25.3) | 20 to 49 observations | 73 (33.9) | 39 (23.4) | 20 to 49 observations |
| Poland | 997 (80.6) | 1095 (88.5) | 165 (13.3) | 94 (8.0) | 268 (23.0) | 246 (26.9) | 176 (27.0) |
| Portugal | 1233 (91.0) | 1275 (94.1) | 462 (34.1) | 409 (32.5) | 456 (36.3) | 396 (42.4) | 222 (32.0) |
| Romania | 534 (83.8) | 581 (91.1) | 55 (8.6) | 20 to 49 observations | 20 to 49 observations | less than 20 observations | 20 to 49 observations |
| Slovakia | 281 (90.0) | 300 (96.1) | 20 to 49 observations | 85 (29.4) | 62 (21.4) | 62 (28.8) | 55 (35.5) |
| Slovenia | 235 (81.0) | 272 (93.7) | 20 to 49 observations | 128 (48.8) | 94 (35.6) | 20 to 49 observations | 20 to 49 observations |
| Spain | 1072 (95.2) | 1062 (94.3) | 554 (49.2) | 129 (12.6) | 247 (24.1) | 239 (31.5) | 132 (22.8) |
| Sweden | 130 (79.7) | 148 (90.8) | 38 (23.2) | 20 to 49 observations | 61 (44.2) | 20 to 49 observations | 20 to 49 observations |
| The Netherlands | 279 (90.5) | 295 (95.7) | 264 (85.7) | 25 (9.0) | 91 (32.9) | 71 (34.4) | 20 to 49 observations |
| United Kingdom | 986 (86.1) | 1026 (89.6) | 989 (86.3) | 420 (41.2) | 351 (34.4) | 180 (24.0) | 132 (22.5) |

†Measurement in the last year ‡ measurement biennial. ¶ At least once in life # Once every three years

Supplement 5: Factors associated with the compliance of each preventive services in women with diabetes (n= 7,353)

|  | **Model 1: Cholesterol measurement**  **OR (95% CI)** | **Model 2: BP measurement**  **OR (95% CI)** | **Model 3: Influenza vaccination**  **OR (95% CI)** | **Model 4: Faecal occult**  **blood testing**  **OR (95% CI)** | **Model 5: Colonoscopy**  **OR (95% CI)** |
| --- | --- | --- | --- | --- | --- |
| **Age**  40-44years | Reference | Reference | Reference |  |  |
| 45-49 years | 1.46 (0.96-2.22) | 0.86 (0.48-1.53) | 1.12 (0.76-1.65) |  |  |
| 50-54 years | 1.76 (1.19-2.60) | 1.09 (0.62-1.89) | 1.26 (0.88-1.79) | Reference | Reference |
| 55-59 years | 2.60 (1.77-3.82) | 1.13 (0.66-1.91) | 1.14 (0.81-1.61) | 1.12 (0.89-1.41) | 1.13 (0.90-1.42) |
| 60-64 years | 2.26 (1.57-3.27) | 1.22 (0.72-2.06) | 1.37 (0.98-1.90) | 1.02 (0.82-1.27) | 1.32 (1.06-1.64) |
| 65-69 years | 2.41 (1.67-3.46) | 1.12 (0.67-1.87) | 2.01 (1.45-2.79) | 0.99 (0.80-1.23) | 1.26 (1.02-1.56) |
| 70-74 years | 2.09 (1.45-3.01) | 1.34 (0.79-2.26) | 2.22 (1.60-3.09) | 0.86 (0.69-1.08) | 1.38 (1.11-1.71) |
| **Educational Level (Low: reference)** | | |  |  |  |
| Middle | 1.12 (0.94-1.34) | 1.57 (1.25-1.95) | 0.75 (0.66-0.84) | 1.33 (1.17-1.52) | 1.20 (1.06-1.36) |
| High | 1.07 (0.81-1.40) | 1.54 (1.08-2.19) | 1.08 (0.90-1.31) | 1.28 (1.03-1.58) | 1.45 (1.19-1.78) |
| **HHI: (1: reference)** 2 | 1.19 (0.96-1.48) | 1.27 (0.96-1.66) | 1.01 (0.87-1.17) | 0.89 (0.75-1.04) | 0.91 (0.77-1.06) |
| 3 | 1.16 (093-1.45) | 1.43 (1.06-1.93) | 0.90 (0.77-1.06) | 0.80 (0.67-0.95) | 0.95 (0.81-1.13) |
| 4 | 1.46 (1.13-1.88) | 1.32 (0.97-1.81) | 0.93 (0.79-1.11) | 0.82 (0.68-0.99) | 1.09 (0.92-1.31) |
| 5 | 0.85 (0.65-1.11) | 1.16 (0.81-1.64) | 1.02 (0.84-1.25) | 0.83 (0.66-1.03) | 0.93 (0.75-1.15) |
| **SPH: (Very good and good: reference)** | |  |  |  |  |
| Fair | 1.04 (0.86-1.26) | 1.27 (1.00-1.61) | 0.82 (0.72-0.94) | 1.03 (0.89-1.20) | 1.01 (0.87-1.16) |
| Bad and very bad | 1.09 (0.86-1.36) | 1.45 (1.08-1.95) | 0.73(0.63-0.86) | 0.95 (0.79-1.13) | 1.24 (1.05-1.46) |
| **Lifestyle Factors** Healthy diet | 1.32 (1.12-1.55) | 1.19 (0.96-1.47) | 1.27 (1.13-1.43) | 1.15 (1.01-1.31) | 1.01 (0.90-1.15) |
| Exercise | 0.89 (0.40-1.99) | 0.99 (0.35-2.77) | 2.09 (1.12-3.90) | 1.02 (0.56-1.87) | 1.53 (0.81-2.86) |
| Tobacco | 0.91 (0.74-1.12) | 0.74 (0.57-0.96) | 0.85 (0.73-1.00) | 0.80 (0.67-0.95) | 0.95 (0.80-1.13) |
| **Chronic conditions**  Obesity | 1.35 (1.15-1.58) | 1.38 (1.12-1.70) | 1.16 (1.04-1.29) | 0.99 (0.88-1.12) | 1.01 (0.90-1.14) |
| Hypertension | 0.85 (0.72-1.00) | 0.58 (0.47-0.71) | 1.04 (0.93-1.16) | 1.06 (0.93-1.20) | 1.18 (1.05-1.34) |
| Coronary heart disease | 1.31 (1.02-1.70) | 1.37 (0.95-1.97) | 0.83 (0.71-0.97) | 0.94(0.80-1.12) | 0.95 (0.81-1.12) |
| Stroke | 1.10 (0.72-1.69) | 0.84 (0.43-1.62) | 1.16 (0.86-1.55) | 0.99 (0.72-1.34) | 1.05 (0.78-1.40) |
| Renal disease | 0.70 (0.51-0.97) | 0.74 (0.48-1.15) | 1.03 (0.85-1.25) | 0.79 (0.64-0.97) | 0.79 (0.65-0.96) |

Supplement 6: Factors associated with the compliance of each preventive services in men with diabetes (n= 7,819)

|  | **Model 1: Cholesterol measurement**  **OR (95% CI)** | **Model 2: BP measurement**  **OR (95% CI)** | **Model 3: Influenza vaccination**  **OR (95% CI)** | **Model 4: Faecal occult**  **blood testing**  **OR (95% CI)** | **Model 5: Colonoscopy**  **OR (95% CI)** |
| --- | --- | --- | --- | --- | --- |
| **Age**  40-44years | Reference | Reference | Reference |  |  |
| 45-49 years | 1.57 (1.01-2.44) | 1.27 (0.76-2.10) | 1.83 (1.21-2.77) |  |  |
| 50-54 years | 1.94 (1.28-2.93) | 1.55 (0.96-2.51) | 1.96 (1.33-2.90) | Reference | Reference |
| 55-59 years | 1.75 (1.19-2.59) | 1.61 (1.02-2.55) | 1.87 (1.28-2.74) | 0.94 (0.75-1.18) | 1.03 (0.83-1.29) |
| 60-64 years | 1.63 (1.12-2.37) | 1.57 (1.00-2.44) | 2.38 (1.64-3.46) | 1.34 (1.09-1.65) | 1.55 (1.26-1.90) |
| 65-69 years | 1.86 (1.27-2.71) | 1.98 (1.26-3.10) | 3.97 (2.74-5.74) | 1.31 (1.07-1.61) | 1.60 (1.31-1.95) |
| 70-74 years | 1.72 (1.17-2.52) | 1.77 (1.12-2.80) | 4.98(3.43-7.23) | 1.22 (0.99-1.50) | 1.93 (1.57-2.37) |
| **Educational Level (Low: reference)** | | |  |  |  |
| Middle | 0.89 (0.73-1.08) | 1.08 (0.86-1.37) | 0.81 (0.71-0.92) | 1.32 (1.15-1.52) | 1.12 (0.98-1.28) |
| High | 1.04 (0.80-1.36) | 1.45 (1.05-2.01) | 1.31 (1.11-1.55) | 1.48 (1.23-1.77) | 1.83 (1.54-2.18) |
| **HHI: (1: reference)** 2 | 1.29(1.02-1.63) | 1.15 (0.87-1.52) | 1.13 (0.96-1.32) | 0.88 (0.73-1.05) | 0.94 (0.79-1.12) |
| 3 | 1.36 (1.08-1.72) | 1.56 (1.16-2.09) | 1.00 (0.85-1.18) | 1.05 (0.88-1.25) | 0.98 (0.83-1.17) |
| 4 | 1.57 (1.23-2.00) | 1.64 (1.21-2.22) | 1.02 (0.86-1.20) | 1.03 (0.86-1.23) | 1.13 (0.95-1.34) |
| 5 | 1.79 (1.37-2.34) | 1.49 (1.09-2.04) | 0.98 (0.82-1.17) | 0.96 (0.79-1.16) | 1.05 (0.87-1.26) |
| **SPH: (Very good and good: reference)** | |  |  |  |  |
| Fair | 1.37 (1.15-1.63) | 1.50 (1.22-1.84) | 0.90 (0.80-1.02) | 1.06 (0.93-1.21) | 1.20 (1.06-1.36) |
| Bad and very bad | 1.25 (1.00-1.57) | 1.87 (1.38-2.53) | 0.90 (0.78-1.05) | 1.09 (0.92-1.28) | 1.23 (1.05-1.45) |
| **Lifestyle Factors** Healthy diet | 1.22 (1.04-1.43) | 1.01 (0.83-1.23) | 1.21 (1.09-1.35) | 1.16 (1.03-1.30) | 1.04 (0.9531.16) |
| Exercise | 0.67 (0.32-1.39) | 0.93 (0.42-2.05) | 1.26 (0.82-1.93) | 0.73 (0.47-1.14) | 1.08 (0.69-1.70) |
| Tobacco | 0.86 (0.71-1.02) | 0.87 (0.70-1.08) | 0.74 (0.65-0.84) | 0.85 (0.74-0.98) | 0.81 (0.70-0.92) |
| **Chronic conditions**  Obesity | 1.18 (1.00-1.39) | 1.29 (1.05-1.58) | 1.04 (0.94-1.16) | 1.02 (0.91-1.14) | 1.11 (0.99-1.24) |
| Hypertension | 0.79 (0.68-0.93) | 0.51 (0.42-0.62) | 0.91 (0.82-1.02) | 0.95 (0.85-1.07) | 0.96 (0.86-1.08) |
| Coronary heart disease | 1.59 (1.22-2.07) | 1.75 (1.21-2.54) | 0.92 (0.80-1.07) | 0.95 (0.82-1.41) | 0.93 (0.81-1.08) |
| Stroke | 0.93 (0.61-1.41) | 0.99 (0.56-1.74) | 1.02 (0.79-1.31) | 1.08 (0.82-1.41) | 1.21 (0.93-1.57) |
| Renal disease | 0.64 (0.45-0.92) | 0.32 (0.17-0.62) | 0.87 (0.72-1.06) | 0.94 (0.76-1.16) | 0.76 (0.63-0.93) |
